# Predicting HLA Class I Non-Permissive Amino Acid Residues Substitutions

**DOI:** 10.1371/journal.pone.0041710

**Published:** 2012-08-08

**Authors:** T. Andrew Binkowski, Susana R. Marino, Andrzej Joachimiak

**Affiliations:** 1 Biosciences Division, Argonne National Laboratory, Midwest Center for Structural Genomics, Argonne, Illinois, United States of America; 2 Computation Institute, University of Chicago, Chicago, Illinois, United States of America; 3 Department of Pathology, University of Chicago, Chicago, Illinois, United States of America; University of South Florida, United States of America

## Abstract

Prediction of peptide binding to human leukocyte antigen (HLA) molecules is essential to a wide range of clinical entities from vaccine design to stem cell transplant compatibility. Here we present a new structure-based methodology that applies robust computational tools to model peptide-HLA (p-HLA) binding interactions. The method leverages the structural conservation observed in p-HLA complexes to significantly reduce the search space and calculate the system’s binding free energy. This approach is benchmarked against existing p-HLA complexes and the prediction performance is measured against a library of experimentally validated peptides. The effect on binding activity across a large set of high-affinity peptides is used to investigate amino acid mismatches reported as high-risk factors in hematopoietic stem cell transplantation.

## Introduction

The human leukocyte antigen (HLA) molecules are highly polymorphic cell membrane associated glycoproteins encoded in a cluster of genes located in the short arm of chromosome 6, the 6p21.1–21.3 region. These molecules play a critical role in adaptive immunity by presenting antigenic peptides to T-cells [1]. T-cells are effector cells of adaptive immunity that have the capability to differentiate self from non-self antigenic peptides bound to HLA molecules. Recognition of foreign peptides, such as those from pathogens or tumors, by T-cells initiates an effector immune response aimed to eliminate harmful cells. In hematopoietic stem cell transplantation (HSCT), immunocompetent donor T-cells may recognize non-self peptides bound to either host matched HLA molecules or to host mismatched HLA molecules and initiate an unwanted immune response against the host, the deleterious process called graft-versus-host disease (GvHD) [2], [3].

There are two major classes of HLA molecules, the class I molecules (HLA-A, B, and C) that are expressed on all nucleated cells and are recognized by CD8+ T-cells and the class II molecules (HLA-DR, DQ, and DP) that are expressed only on antigen presenting cells and are recognized by CD4+ T-cells. Extensive HLA polymorphism, different versions or alleles of each of the HLA molecules within the population, in an individual ensures binding of diverse antigenic peptides for presentation to the immune system [1]. HLA class I ligands are comprised of proteolysed protein fragments, between 8–12 amino acids in length, derived from endogenous proteins that are degraded by cytosolic proteinases. Once bound, the peptide-HLA (p-HLA) complex is transported to the cell surface and presented for recognition by the T-cell receptors of CD8+ cytotoxic T-cells. While nonameric peptides (with amino acid positions defined as P1–P9) have been shown to bind preferentially, peptides between 8–12 amino acids of length can also bind to HLA class I molecules. Longer peptides are accommodated in the groove by adopting a dramatically more bowed conformation.

**Figure 1:**
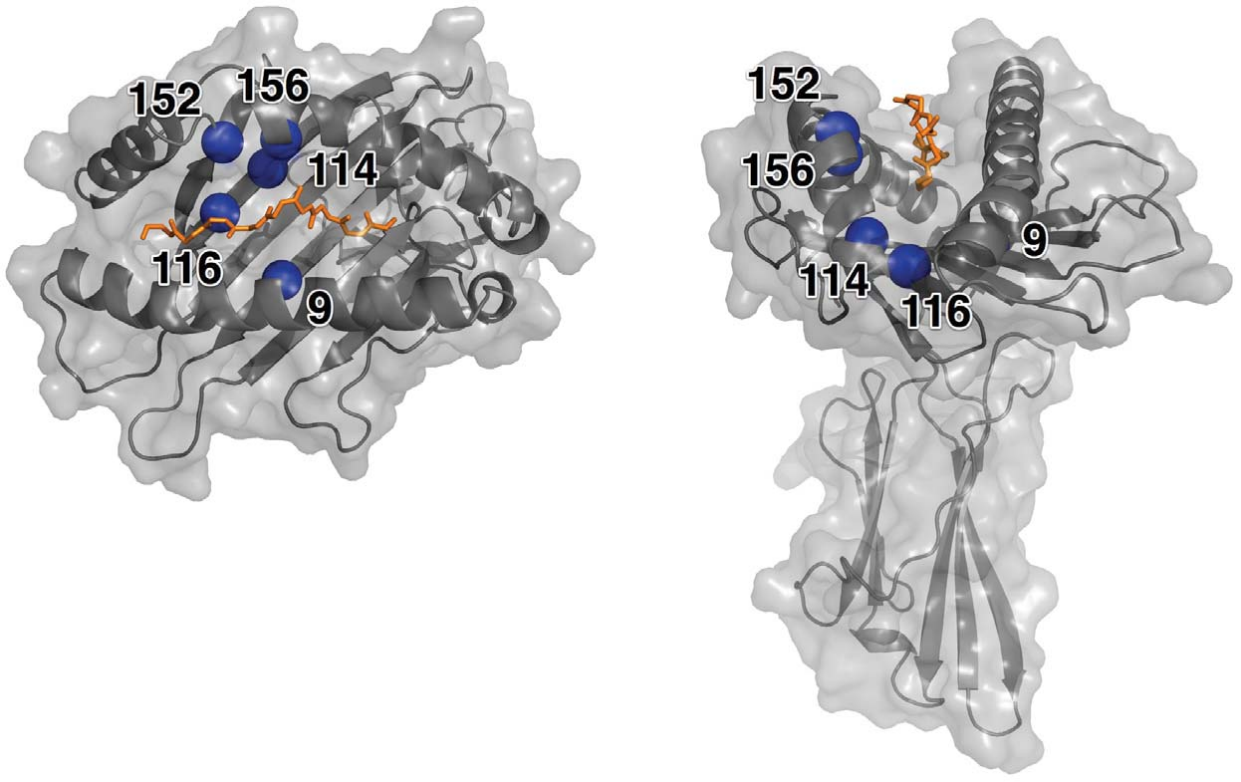
Structure of the HLA*A2∶01 Molecule. The structure of the HLA-A*02∶01 (PDB id = 1AKJ) molecule shown with bound peptide backbone (chain C, orange). The positions of the five high-risk residues on the HLA molecule are represented by a blue sphere and labeled. The molecular surface of the molecule is shown in gray.

The HLA class I peptide-binding groove is a well-defined cavity formed between the α-helices of the α1 and α2 domains and by a six member anti-parallel β-sheet that comprises the floor. In the HLA-A*02∶01 allele, the binding groove is delineated by 37 residues (residue numbers 5, 7, 9, 26, 45, 58, 59, 62, 63, 66, 67, 69, 70, 73, 74, 77, 80, 81, 84, 97, 99, 114, 116, 123, 124, 133, 143, 146, 147, 152, 155, 156, 159, 163, 164, 167, 171) with varying degrees of solvent accessibility [4]. The groove cavity spans approximately 32 Å, providing a physical limitation to the length of bound peptides. Individual HLA allele sequence variability results in measurable differences in the groove’s solvent accessible surface area and volume. As a reference, the surface area and volume for the HLA-A*02∶01 allele (PDB id = 1AKJ [5]) are 425.9 Å^2^ and 546 4 Å^3^, respectively [4]. The structure of a HLA-A*02∶01 allele with bound peptide ligand (PDB id = 1AKJ) is shown in Figure 1.

The polymorphic nature of the HLA molecules ensures that a wide variety of peptides originating from proteins of invading pathogens can bind with sufficient affinity to satisfy their role in the triggering of the immune system response cascade. This is accomplished through a combination of amino acid sequence dependent and independent interactions [6]. Structural analysis of existing p-HLA complexes in the Protein Data Bank (PDB) have revealed common features of interaction. The most notable sequence independent interactions are derived from hydrogen bonds with the amino and carboxyl groups at the N and C termini, while sequence dependent interactions are distributed along the length of the binding groove in pockets (designated A, B, C, D, E and F) that contribute to allele-specific peptide recognition and specificity [7], [8]. The peptide primary anchor residues, providing the main contact points, are located at residue positions P1, P2 and P9. Once fixed at these positions, the remaining peptide residues assume complementary positions in the groove, contributing additional, albeit less significant, interactions.

While HLA polymorphism provides a broad response mechanism, the diversity of candidate peptides capable of binding to HLA molecules makes exhaustive experimental studies prohibitive. An attractive alternative is an immunoinformatics approach utilizing computational models to predict p-HLA binding. For well-characterized HLA alleles, supported by high-quality experimental data, it is possible to computationally predict binding using the peptide’s amino acid sequence. Initially, sequence motifs [9], [10] and matrix-based models [11], [12], [13] were used. More recently, advanced machine-learning techniques utilizing Hidden Markov Models [8], [14], [15], [16], artificial neural networks [15], [17], [18] and support vector machines [19], [20], [21] have been applied. Many sequence-based methodologies have excellent predictive abilities [16], however, their application is strictly limited to alleles with sufficient experimental training data (e.g., HLA-A*02∶01, HLA-A*01∶01, HLA-B*07∶02, etc.). Unfortunately, experimental data is not available for many alleles and new alleles are continuously being discovered [22]. In addition, these methods do not provide specific models for interactions that may be critical to understand peptide binding or to interpret prediction results.

Structure-based binding prediction methods have the potential to overcome the limitations and challenges of sequence-based methods [23], [24]. There are nearly 400 crystal structures of HLA molecules available in the PDB (November 8, 2011) [25], providing high-quality structures as input for modeling. Structure-based methods, however, confront their own constraints due to the implementation complexity and the high computational costs. Search of a large sequence database is typically easily accomplished but can be impracticable for the structure-based methods due to the prohibitive runtimes. Structure-based methods share the need to identify a suitable pose for a peptide molecule into the HLA molecule, requiring an expensive conformational space search for both HLA and peptide molecules. Different approaches have been developed using homology modeling [23], [26], [27], [28], [29], threading [30], [31], and docking [23], [28]. In practice, many of these methods combine multiple techniques, for example Tong *et al.* [23] and Kumar *et al.* [28] combined docking and homology modeling. Once a peptide is posed, a scoring function is applied to make a quantitative evaluation of the binding based on the atomic interactions with the HLA molecule. Scoring functions have been applied based on potential matrix based pairs [28], [32], semi-empirical methods [29], [33], [34], and quantitative-structure-activity-relationship methods [35], [36]. The treatment and parameterization of the p-HLA system influence both the accuracy and runtime. For example, some methods use solvent in their prediction algorithm [34], [37], while others ignore its role [6], [23], [38] .

Clearly, there is a need for the development of more accurate approaches to predict peptide binding in a wide spectrum of clinical applications from vaccine design to the understanding the molecular basis of allorecognition in HSCT. For a predictive method to be broadly applicable, it must reduce reliance on large sets of experimental data and should be efficient. Here, we introduce a methodology using robust computational approaches to model protein-ligand interactions. The method combines peptide modeling, docking and physics-based scoring within a highly scalable supercomputing environment. The method is applied to predict the effect on binding activity across a large set of high-affinity peptides to investigate non-permissive amino acid mismatches reported as high-risk-factors in HSCT.

## Results

### Assessing Structural Conservation and Variability of the HLA Binding Groove

To explore the structural variations of peptides within the binding groove, a structural superimposition of 50 HLA-A*02∶01 crystal structures in complex with 50 distinct peptides from the PDB was performed (see Methods). The peptide chains were excluded from the molecule during the structural alignment procedure. The mean root mean square deviation (RMSD) between HLA structures is 0.69 Å for the backbone and 0.72 Å for the all-atom alignment. Considering only the 37 solvent accessible residues comprising the binding groove (Figure 1) [4] the mean RMSD is 0.26 Å for the backbone and 0.27 Å for the all-atom case.

**Figure 2:**
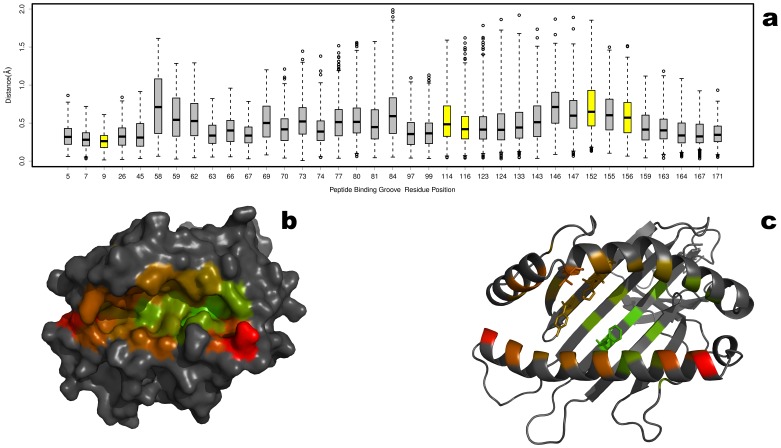
Conservation of the HLA Peptide Binding Groove. The structural conservation of the peptide binding groove was measured by performing a superposition of 50 unique p-HLA complexes from the PDB. The pairwise RMSD was calculated between the backbone atoms at each solvent accessible residue comprising the binding groove [4]. The results are summarized as a boxplot showing the median, quartiles, maximum and minimum distances, and outliers (circles) at each residue position. The colors are scaled from green to red (lowest to highest RMSD) with minimum values of 0.28 Å (residue 9) and maximum of 0.83 Å (residue 58). For contrast, the non-binding groove residues are colored black. The average RMSDs are color mapped on to the HLA molecule shown as surface representation (b) and cartoon representation (c) from green (low RMSD) to red (high RMSD).

The pairwise RMSD (backbone) between all 50 structures was calculated for each binding groove residue position and is presented as a boxplot in Figure 2a. Across the entire binding groove, the average RMSD (backbone) at each position is less than 0.83 Å, indicating an extremely high level of structural conservation of the HLA molecule. The averaged RMSD values are mapped onto depictions of the HLA model shown in surface representation and cartoon representation (Figure 2bc). Given that each structure in the data set has a unique peptide bound, the HLA molecule affords very limited structural accommodations for peptide binding. This agrees with the previously reported limited induced fit of the HLA molecule in response to binding peptides with different sequences [6], [39].

### Structural Variability of Bound Peptides

**Figure 3:**
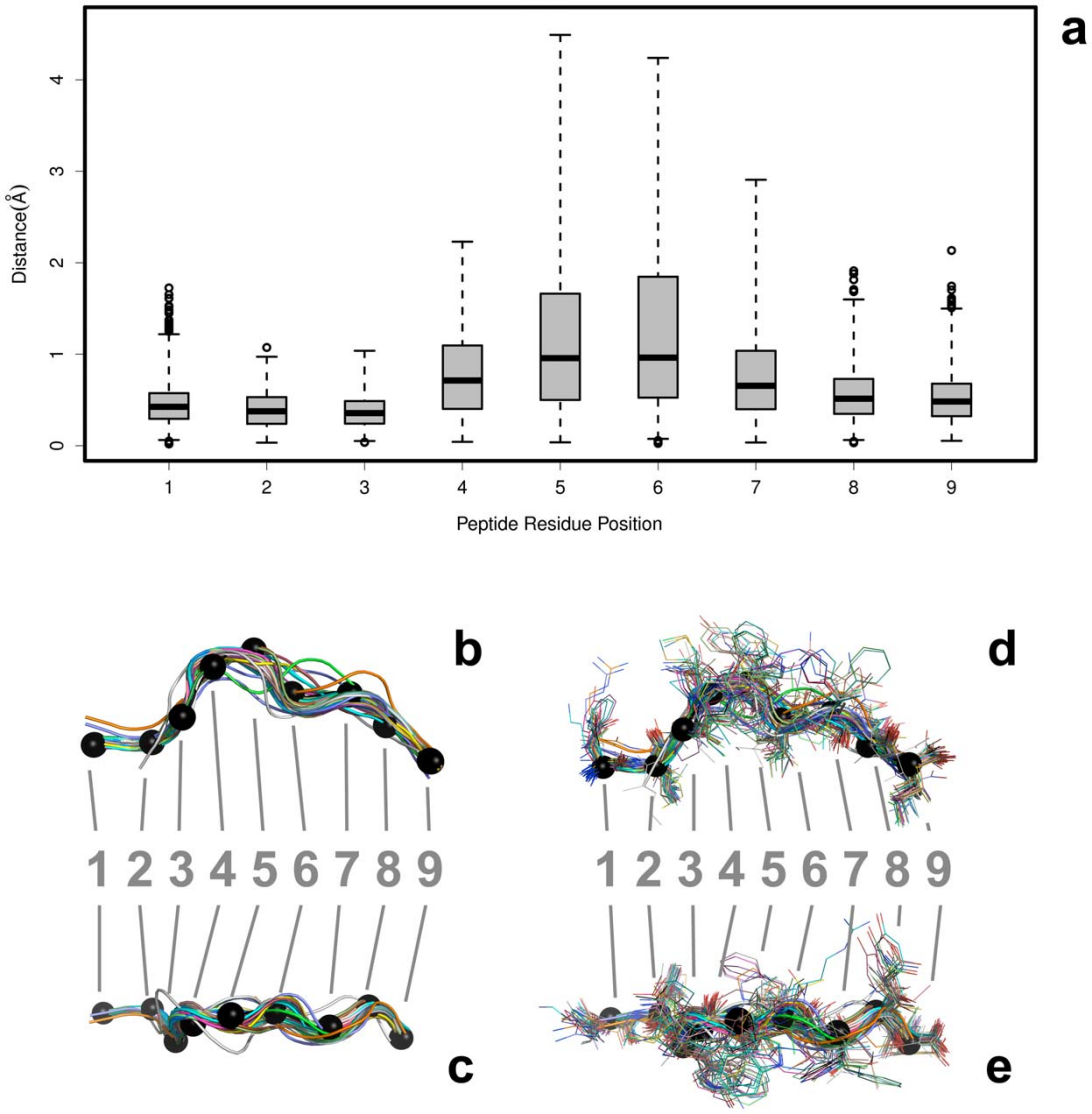
Variability of Peptides Bound to HLA Molecules. The structural variability at each residue position for bound nonameric peptides in p-HLA complexes from the PDB. After a structural alignment of the HLA molecules, the peptide coordinates were extracted. The aligned peptides are depicted from the side view (b and d) and top (looking down into the binding groove) view (c and e), both with and without side chains. The backbone-only models are shown in B (side view) and C (top-down view). The alignments with the side chains are shown in D (side view) and E (top-down view). The Cα atoms are shown as black spheres. The pairwise RMSD was calculated between the backbone atoms at each residue position for each peptide. The peptides are colored uniquely and, for reference, the Cα atoms from a peptide (PDB id = 1AKJ, chain = C) are shown as black spheres. The results are summarized as a boxplot showing the median, quartiles, maximum and minimum distances, and outliers (circles) at each residue position.

The bound peptide conformations were analyzed by replacing the peptide chains back into the reference frames of their aligned HLA counterparts (Figure 3). The pairwise RMSD was calculated between the backbone atoms at each residue position and the results are shown as a boxplot in Figure 3a. While the average RMSD at each position of bound peptide for the different HLA-A*02∶01 molecules is less than 1.0 Å, the range variance observed for the middle residues is significantly greater. This is in agreement with the previously reported observations of peptide binding interactions: the termini and anchoring residues occupy highly fixed positions, while the interior residues show flexibility that allow them to lessen unfavorable side chain interactions with the HLA molecule and solvent [23], [38], [39], [40]. This peptide flexibility accounts for the canonical “bow” shape seen in several bound peptide conformations (Figure 3b and 3d).

The set of HLA-bound peptides mainly represents well-characterized antigens and their sequence bias may explain some sequence preference and structural conservation. We analyzed the amino acid sequences of bound peptides (Figure S1) and have concluded that, with the exception of positions P2 and P9, the current set of peptides is not significantly biased. Therefore for all structurally characterized p-HLA complexes, both the main chains of the HLA-A*02∶01 molecule and a bound peptide are highly structurally conserved.

### Modeling Bound Peptides in HLA-A*02∶01 and Benchmarking

The computational complexity required to accurately model and score bound peptide arises from two components: the vast conformational space must be adequately sampled to identify a suitable pose and application of intricate algorithms used to score poses. Our new procedure overcomes these constraints by imposing selective structural conservation to reduce the conformational search space and by using highly-scalable methodologies, implemented in a supercomputing environment, to calculate binding free energy using the MM-GBSA (molecular mechanics/generalized Born surface area) method. We utilize a template-based methodology based on consensus anchoring coordinates interpolated from structural data (see Methods). A given peptide backbone can be positioned into the binding groove through an initial coordination of the termini and anchoring positions (P1, P2 and P9). The side chains are assigned to the backbone using a multi-scale potential function [41].

We assessed our procedure by “redocking” peptides from p-HLA crystal structures. For each complex, we extracted the coordinates of the peptide from the complex and then docked it back into the HLA-A*02∶01 molecule. After the initial placement, the resulting peptide was treated as fully flexible and underwent a refinement procedure to finalize its pose. The RMSD measurement was then calculated between the experimental and redocked pose coordinates of the peptide. An RMSD was also calculated between the overall complex (i.e., the HLA and peptide molecules) to assess conformational changes in the HLA molecule. The results for the data set are summarized in Table 1.

**Table 1:** Peptide-HLA Molecule Modeling Benchmark.

PDB	Resolution	Sequence	RMSD		RMSD	
Code			Complex	Backbone	Complex	Backbone
1AKJ	2.65	ILKEPVHGV	1.64	0.94	1.61	0.82
1AO7	2.6	LLFGYPVYV	1.64	0.74	1.8	1.14
1B0G	2.5	ALWGFFPVL	1.6	0.8	1.83	1.00
1EEY	2.25	ILSALVGIV	1.54	0.87	1.54	0.94
1EEZ	2.3	ILSALVGIL	1.65	1.18	1.65	1.11
1HHG	2.6	TLTSCNTSV	1.55	0.91	1.69	1.24
1HHI	2.5	GILGFVFTL	1.54	0.76	1.73	1.03
1I1F	2.8	FLKEPVHGV	1.69	1.07	1.69	0.94
1I1Y	2.2	YLKEPVHGV	1.65	0.93	1.71	0.98
1I7R	2.2	FAPGFFPYL	1.66	1	1.89	1.04
1I7T	2.8	ALWGVFPVL	1.63	1.03	1.76	1.04
1I7U	1.8	ALWGFVPVL	1.62	0.91	1.76	1.08
1JHT	2.15	ALGIGILTV	1.51	0.87	1.56	1.06
1QEW	2.2	FLWGPRALV	1.63	0.86	1.86	1.13
1QR1	2.4	IISAVVGIL	1.52	0.88	1.59	1.05
1QRN	2.8	LLFGYAVYV	1.63	0.81	1.83	1.29
1QSE	2.8	LLFGYPRYV	1.72	0.96	1.81	1.24
1QSF	2.8	LLFGYPVAV	1.56	0.68	1.79	1.09
1S8D	2.2	SLANTVATL	1.58	0.98	1.57	1.06
1S9W	2.2	SLLMWITQC	1.67	0.93	1.76	1.17
1S9X	2.5	SLLMWITQA	1.66	0.9	1.74	1.17
1S9Y	2.3	SLLMWITQS	1.65	0.87	1.76	1.14
1T1W	2.2	SLFNTIAVL	1.58	0.85	1.72	1.19
1T1X	2.2	SLYLTVATL	1.65	0.99	1.67	1.10
1T1Y	2	SLYNVVATL	1.6	0.94	1.7	1.10
1T1Z	1.9	ALYNTAAAL	1.58	0.95	1.58	0.99
1T20	2.2	SLYNTIATL	1.64	1.02	1.7	1.17
1T21	2.19	SLYNTVATL	1.6	0.86	1.71	1.18
1TVB	1.8	ITDQVPFSV	1.63	0.84	1.7	1.08
1TVH	1.8	IMDQVPFSV	1.65	0.82	1.72	1.11
2BNQ	1.7	SLLMWITQV	1.71	0.89	1.65	0.98
2GIT	1.7	LLFGKPVYV	1.84	0.84	1.91	0.99
2GTW	1.55	LAGIGILTV	1.64	0.91	1.94	1.53
2GUO	1.9	AAGIGILTV	1.51	0.93	1.58	1.09
2V2X	1.6	SLFNTVATL	1.57	0.76	1.66	1.12
2X4O	2.3	KLTPLCVTL	1.88	1.53	1.74	1.22
2X4R	2.3	NLVPMVATV	1.93	1.66	1.88	1.68
2X4S	2.55	AMDSNTLEL	1.69	1.06	1.67	1.06
3D25	1.3	VLHDDLLEA	1.7	1.09	1.63	1.02
3FQT	1.8	GLLGSPVRA	1.73	1.34	1.79	1.38
3FQW	1.93	RVASPTSGV	1.69	1.18	1.87	1.48
3FT4	1.9	VLRDDLLEA	2.41	2.33	2.34	2.23
3GSQ	2.12	NLVPSVATV	1.65	1.22	1.61	1.18
3GSR	1.95	NLVPVVATV	1.66	1.12	1.69	1.24
3GSU	1.8	NLVPTVATV	1.71	1.25	1.72	1.34
3GSV	1.9	NLVPQVATV	1.71	1.17	1.69	1.16
3GSW	1.81	NLVPMVAAV	2.49	2.46	2.27	2.23
3GSW	1.81	NLVPMVAAV	1.49	1.46	1.27	1.23
3GSX	2.1	NLVPMVAVV	1.72	1.19	1.68	1.16
3H7B	1.88	MLWGYLQYV	1.85	1.36	1.93	1.33
3KLA	1.65	SLLMWITQL	1.79	1.19	1.75	1.21

A set of 50 unique p-HLA complexes from the PDB was used to benchmark our modeling methodology. For each complex, the bound peptides were removed and then redocked back in to the HLA molecule. The RMSD between our solution and the experimental model is shown for the all-atom complex and the backbone-only atoms. Using each sequence, an *ab initio* three-dimensional model of each peptide was constructed. The *ab initio* peptide was then docked to the HLA molecule. The RMSD between our solution and the experimental model is shown for the all-atom and backbone-only complexes.

All peptides in our data set could be posed within the 2.5 Å RMSD threshold for a “correct solution” as proposed by Tong and collaborators for p-HLA complexes [23]. Our procedure posed 30 out of 50 peptides within 1.0 Å of the experimental model. Of the remaining 20 peptides, 19 were less than 1.7 Å of their experimental models. The only marginal performer at the 2.5 Å cutoff was HA-1^Arg^ peptide (*VLRDDLLEA*; PDB id = 3FT4), a non-immunogenic variant of the highly immunogenic HA-1^His^ peptide (Text S1). Measuring the overall RMSD of our docked p-HLA complex to the original experimental complex, 48 of the 50 complexes were within 2.0 Å distance. This agreement provides confidence that our docking procedure is robust and does not require significant rearrangement or disruption of the HLA molecule and peptide. We conclude that our method performs at least as well as previously reported p-HLA redocking studies [23].

### *Ab Initio* Modeling Peptides and Docking to HLA

The high degree of structural conservation observed for bound peptides (Figure 3) also provides a useful scaffold for *ab initio* peptide modeling. This allows for the generation of three-dimensional peptide models using only a given amino acid sequence as input, providing the opportunity to probe HLA interactions with any peptide presented to the human immune system. Our analysis revealed that accurate docked peptide poses were highly dependent on the modeling of the N-terminal residue side chain. That is, if the P1 residue was correctly modeled the remaining side chains were more likely to be posed correctly. Therefore, we prepared a series of N-termini specific templates from the existing p-HLA complex structures to aid modeling (see Methods).

Our procedure was tested on 50 peptides from p-HLA complexes with known crystal structures. The all-atom and backbone RMSDs were measured between the *ab initio* and the crystal structure. The backbone RMSDs ranged from 0.20−2.21 Å with a mean value of 1.11 Å. For the all-atom models, the RMSDs ranged from 0.77−2.24 Å with a median value of 1.37 Å. In a few cases, our method was not able to identify the correct rotamer for each peptide and contributed to the overall dissimilarity (Figure S2).

*Ab initio* peptide models derived from exiting p-HLA structures were used to dock to HLA molecules. The backbone and all-atom RMSDs were calculated between the peptide from the docked solution and the original crystal structure. The results are summarized in Table 1. With the exception of HA-1^Arg^, all docked solutions have RMSDs below 1.68 Å for the backbone and 1.96 Å for the all-atom comparisons. Unexpectedly, 15 of the poses generated from the *ab initio* peptides had better RMSDs than those that were simply extracted from the experimental complex and redocked back in. Upon inspection, some side chains from the crystal structures were modeled in energetically strained conformations or showed unusual rotamers. Our method’s ability to find a more favorable side chain arrangement is most likely due to the flexibility of both the peptide and HLA molecule in our docking procedure.

### Scoring Peptide Binding

The Immune Epitope Database (IEDB) is a repository that collects and organizes data on major histocompatibility complex (MHC) binding experiments [42] and has been used to develop and assess computational methods for predicting peptide binding to MHC molecules [16], [39], [43]. The IEDB provides binding affinity measurements as IC50 (and/or EC50) values.

The subjectivity of threshold selection can make the binding classification inconsistent, especially when dealing with a combination of quantitative and qualitative data (see Methods). In addition, interpreting the influence of weak binding peptides in a computational model introduces more complexity when trying to correlate experimental data to computational predictions. We believe that these difficulties are successfully addressed by rationalization of a peptide-binding event. As previously noted by Hou *et al*. [44], in the context of the peptide recognition domains, precise, quantitative binding affinity estimates may not be required to accurately model the systems behavior. The use of a Boolean “binding/no-binding” classification may be adequate to infer whether the immune response cascade escalates to T-cell activation. In the context of our model, this allows us simply to predict if a peptide would bind or not bind to the HLA molecule.

**Figure 4:**
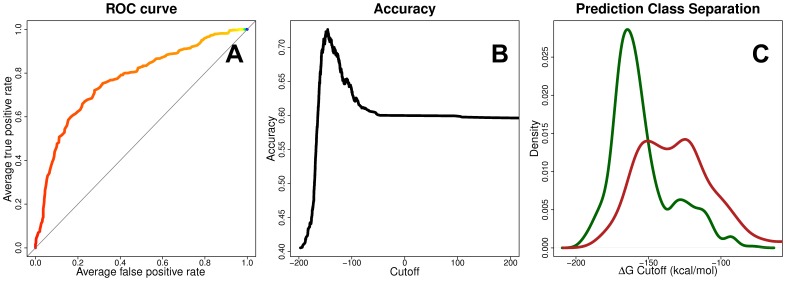
Peptide Binding Prediction Performance. The performance of our docking methodology to predict binding peptides is measured using a subset of nearly 6,000 peptides from the IEDB repository of HLA-A*02∶01 epitope binding affinity data. For our approach, the area under the ROC curve is 0.771 (A). The prediction accuracy is measured at varying cutoff thresholds of calculated ΔΔG values (B). The distribution densities of the calculated ΔΔG values for the positive (green) and negative (red) peptides are shown (C).

A subset of the IEDB database was constructed by removing duplicate entries and data that could not be reconciled (see Methods) and were divided into two definitive groups: binders (IC50≤500 nm) and non-binders (IC50>500 nm) with 2,660 and 3,294 members, respectively. *Ab initio* models were generated for each peptide, docked into HLA-A*02∶01, and the binding free energy of the peptide was calculated. The overall performance of our prediction methodology is evaluated by the ability to distinguish binding from non-binding peptides (see Methods), and is evaluated by a receiver operator characteristic (ROC) curve (Method S3). The prediction accuracy of our method, as measured by the area under the ROC curve (AUC), is 0.771 (Figure 4).

### Rediscovery of Non-Permissive HLA Residue Substitutions in HSCT

Our *ab initio* modeling of peptide and HLA-A*02∶01 complexes depends solely on a peptide sequence as input and should distinguish binder from non-binder peptides. To assess whether our method is sensitive to amino acid substitutions between mismatched patient and donor HLA alleles that are known to be high-risk, factors in HSCT, we conducted large-scale docking studies against a pool of high-affinity peptides. To date, five positions in the HLA-A peptide-binding groove (9, 114, 116, 152, and 156) have been identified as being high-risk non-permissive substitutions in HSCT (Table 2) [45], [46], [47], [48]. These positions are highlighted in Figure 1.

**Table 2:** Reported High-Risk Amino Acid Residue Substitutions.

Residue Position	Groove Location	Peptide Contacts	Observed Residues	Literature Reference
9	Floor	P2	YTS**F**H	Kawase, 2007; Kawase, 2008; Marino, 2012
114	Sidewall	P5,P6,P7	Q**H**PEDTRSN	Marino, 2012
116	Floor	P9	**Y**HTFSDNV	Ferrara, 2001; Kawase, 2007; Marino, 2012
152	Mouth	P7	E**V**RWMA	Marino, 2012
156	Mouth	P3	W**L**QSRAG	Marino, 2012

Non-permissive residues between patient and donor HLA antigens or alleles reported to have deleterious outcomes in HCT [45], [46], [47], [48]. The observed residues from all known HLA-A alleles at each position are listed as along with their relative orientation in the binding groove. The residue types were obtained from sequence alignments from the IMGHT using A*01∶01∶01∶01 as the reference sequence. The peptide residues in proximity for making contacts to HLA are listed for each residue.

Protein sequences of all known HLA alleles are available through the International Immunogenetics Information System (IMGT)/HLA Database (http://www.ebi.ac.uk/imght/) [5]. A sequence alignment was performed including all HLA-A alleles (IMGT/HLA Release 3.0.0) and the observed residues at each non-permissive position were identified (Table 2). For example, at position 9 the residues threonine (T), serine (S), tyrosine (Y), phenylalanine (F) and histidine (H) are observed. Homology models for each observed residue at each position were generated, resulting in the formation of 35 unique structural models.

For each of the 2,660 high-affinity binding peptide sequences in the IEDB, we constructed an *ab initio* model and docked it to the HLA-A*02∶01 reference molecule. Next, we docked the peptide into each HLA molecule with a non-permissive amino acid substitution. The difference in the estimated binding free energy (ΔΔG) between the reference and substituted molecules was calculated. A negative ΔΔG would have higher predicted binding affinity resulting from the substitution and vice versa. Using an established binding threshold, we predicted whether the substitution would result in loss of binding activity for a peptide.

**Figure 5:**
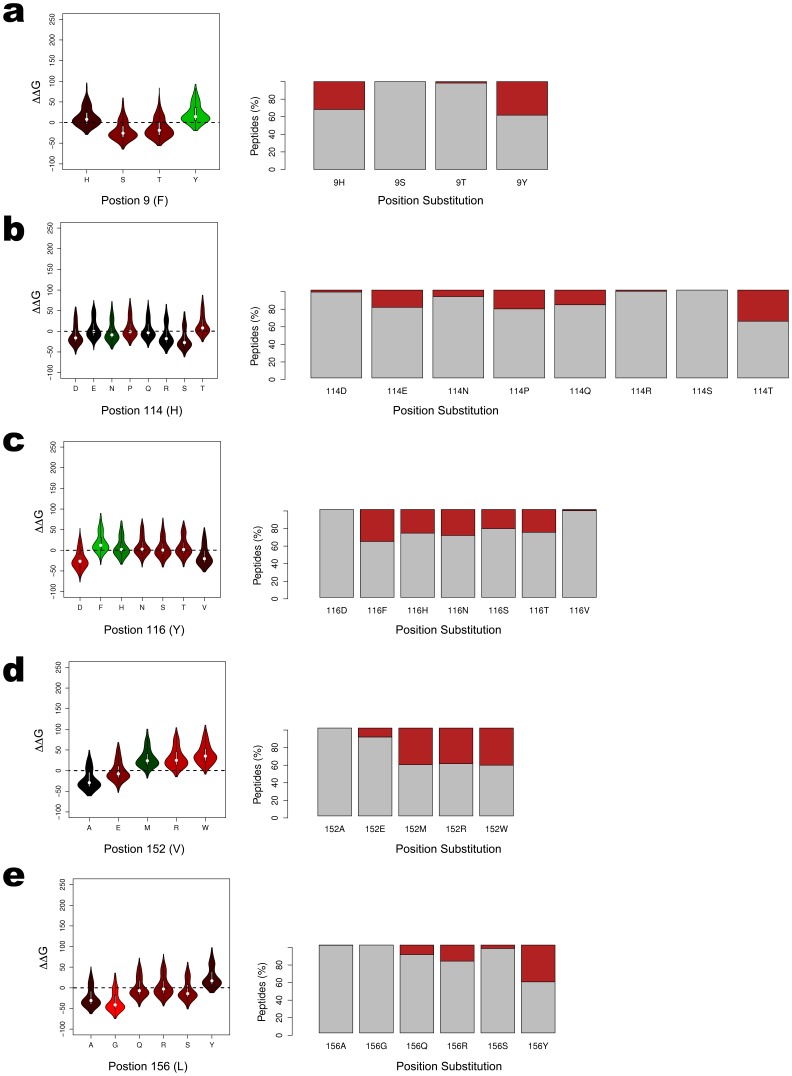
Effect of High-Risk Amino Acid Substitutions on Binding Predictions. The distribution of calculated ΔΔG values for each of the 35 structural models from the five non-permissive substitutions are shown as violin plots. The plots are colored according to the Blosum62 amino acid substitution matrix, typically used for scoring evolutionary divergent protein sequences based on local alignment [52]. The colors are scaled according to the matrix’s log odds values, with green representing high frequency substitutions and red representing low frequency substitutions. Each plot highlights the percentage of peptides that are predicted to no longer bind as a result of the substitution in red. Those peptides predicted to retain their binding activity remain in gray.

The distribution of calculated ΔΔG values for each substituted model is summarized as violin plots in Figure 5. The binding behavior is summarized as a boxplot, highlighting the percentage of peptides that are predicted to no longer bind as a result of the substitution (red) and the percentage of those peptides predicted to retain their binding activity (gray). It should be noted that a suitable pose failed to be produced for only 23 peptides across all 93,100 docking simulations. Irreconcilable steric clashing between the large side chains in the HLA molecule and the consecutive, extended side chain groups from the peptide, were responsible.

Measuring loss of peptide binding activity due to amino acid substitution presents structural evidence for the basis of the reported high-risk behavior. For each position, we observe marked changes in the ability to bind the same peptides in the pool. The residue-level resolution of our modeling allows the specific substitutions resulting in significant loss of predicted binding to be identified. The most notable being position 9 F-Y (34%), 114 H-T (37%), 116 Y-F (32%), 152 V-W (41%), and 156 L-Y (42%). As each substitution may represents a different HLA allele, comparing the repertoire of peptides bound by different alleles may be useful for assessing histocompatibility between patients and donors. For example, the 9 F-Y substitution can be directly mapped to the HLA*02∶06 allele, which differs from the HLA*02∶01 allele only by this substitution. As reported by Kawase *et al* and Marino *et al*, the A:02∶01–A:02∶06 mismatched pair is a significant risk factor for acute GvHD [47] and death at day 100 post-HSCT [48].

## Discussion

Accurate modeling of peptide-protein interactions is a challenging problem. The majority of well-established docking methodologies are developed for predicting small-molecule and protein interactions useful in computer aided drug discovery. As such, many of them are not parameterized or even applicable to docking peptides. Immunogenic peptides are significantly larger and more flexible than “drug-like” compounds, further contributing to the difficulties in working with them. Despite these limitations, new methods have been applied to dock peptides into HLA molecules.

The particular advantage of the p-HLA system is the high degree of structural conservation in a bound complex. The peptide’s main chain atoms adopt a similar position within the groove, which constrains the side chains into a relatively limited number of positions. This orientation preserves the key hydrogen bonding interactions at the main chain termini. We have utilized this conservation to reduce the conformational space search, simplifying the initial peptide placement. Successful docking is, therefore, highly dependent on accurately positioning the less-constrained elements in the complex (e.g., the middle loop and side chains). Our approach is to construct the peptide in the presence of a consensus HLA molecule and to assign the remaining atom position based on hydrogen bond matching and surface shape complementarity. Once fully constructed, the p-HLA complex is allowed to be fully flexible for further minimization and scoring. Benchmarking studies show that our method can faithfully recreate models for existing nonameric peptides and reproduce the poses of existing p-HLA crystal structures from the PDB.

Our choice of the MM-GBSA method for binding free energy estimation represents a reasonable compromise between runtime and accuracy. MM-GBSA has the advantage of better convergence properties and has been successfully applied to the study of major histocompatability complexes [49]. It requires a significant amount of computation time, but can be implemented in a highly scalable approach within a supercomputing environment. Our implementation on the BlueGene/P supercomputer at the Argonne Leadership Computing Facility (Argonne National Laboratory, Argonne, IL, USA) has allowed us to conduct one of the largest applications of MM-GBSA to study p-HLA interactions to date.

Using binding free energy differences as a predictor of binding activity, our method was able to correctly predict binding behavior for 77.1% of a nearly 6,000 peptide dataset. Two other studies have reported *ab initio* structure-based predictions for experimental HLA-A*02∶01 binding data [38], [39]. Bordner and Abagyan, using a considerably smaller and less diverse database (304 binders and 304 non-binders), reported an AUC value of 0.830 [38]. This study is not directly comparable to a pure structured-based method, as a SVM trained model was included in their approach. In addition, the non-binder data set was not based on experimental data, but it was constructed *in silico* by shuffling the sequences of the binding peptides. More recently, Kumar and colleagues reported an AUC of 0.742 for a set of just over 300 nonameric peptides from the IEDB [39]. The better performance of our prediction is further signified by the 20 fold larger dataset that we used.

A distinct advantage of our approach is that it was implemented in a large-scale computing environment allowing for the use of advanced simulation methods that would be prohibitive to execute elsewhere. It also allowed us to carry out our study on a large scale, permitting the inclusion of peptides that may otherwise have been omitted due to limited resources. For example, the 114 H-T substitution can be mapped to the rare HLA-A*02∶94 allele. Such a new and/or rare allele may not be tackled using other predictive methodologies, whereas our approach can provide an accurate initial characterization. In this case, we directly observe that H-T substitution results in the most significant loss of binding at position 114.

In this implementation, we have excluded explicit water molecules from our modeling procedure. While common practice in many docking protocols (especially in the p-HLA systems [6], [23], [38]), the influence and importance of solvent cannot be underestimated [50], [51]. Decisions on the treatment of solvent will affect the outcome of simulations and are complicated by the varying roles that solvent plays mediating and contributing to interactions in the p-HLA system. Our treatment of solvent is based on our aim to develop a general methodology applicable to large-scale peptide libraries for which allele and peptide combinations may not be fully studied by high-resolution X-ray crystallography, leaving the location and role of water molecules unresolved. We will continue to evaluate our treatment of solvent and its affect on performance and runtime in future iterations of our methodology.

Forward looking, our approach allows for the incorporation of additional computationally intense tasks to be modularly included in the computing pipeline. For example, using genome-wide epitope scans as input into the modeling simulations or extending the pipeline to model p-HLA interactions with the T-cell receptor. In concert, these methods may be useful to characterize additional p-HLA complex interactions that may influence peptide binding in a wide range of clinical entities such as vaccine development, immunotherapy against infectious pathogens, HLA associated diseases, autoimmune diseases, pharmacogenomics, as well as to further study high-risk amino acid mismatches in HSCT.

## Methods

### HLA-A*02∶01 Structures from the PDB

The sequence of the HLA-A*02∶01 allele, identified from the IEDB and Analysis Resources (http://www.immuneepitope.org) [42], was used to query all available structures from the PDB (April 13, 2010 release). A BLAST (Basic Local Alignment Search Tool) search identified 113 HLA-A*02∶01 structures. Of those, 111 were solved with bound peptides fragments varying in length from 8 to 10 amino acids. After removing molecules containing gapped peptides (i.e., missing residues), unnatural (modified) residue substitutions, or incompletely modeled side chains, 96 complexes remained. Our final data set was reduced to 50 complexes after removing duplicate peptides and limiting the peptide length to 9 amino acids. The final set of HLA-A*02∶01 peptide complexes are listed in Table 1.

Structural alignment of HLA-A*02∶01 molecules was done using the PyMOL molecular visualization and modeling software (The PyMOL Molecular Graphics System, Version 1.3, Schrödinger, LLC). The alignment was conducted by superimposing the models based on matched atoms between molecules. The bound peptide chains were excluded from consideration during the alignment procedure.

### IEDB Peptide Binding Data

The IEDB was data mined on April 24, 2010 from IEDB to extract HLA-A*02∶01 binding data [42]. The database was filtered to remove ambiguity (Method S1) resulting in 3,294 negative and 2,660 positive peptides.

### Docking Procedure

The docking procedure takes advantage of the structural conservation observed in the p-HLA complex in order to identify an initial pose that will require minimal adjustments. A given peptide is first positioned into the binding groove by superimposing the P1, P2, and P9 residues onto a consensus peptide backbone created by averaging the coordinates of the backbone atoms at these positions from the available crystallographic models. After the initial placement, the side chains are then added to template position utilizing the homology modeling approach to protein side chain assignment as implemented in the application SCWRL [41]. SCWRL uses a potential function based on a combination of factors, including a backbone-dependent rotamer library, a fast anisotropic hydrogen bonding function, a short-range, soft van der Waals atom-atom interaction potential. In our implementation, we included a reference frame consisting of an HLA-A*02∶01 backbone-only structure for additional steric clashing checks.

Once all side chains are assigned, a fast rigid body position refinement is conducted to maximize the hydrogen bond interactions and avoid any large steric hindrance. The displacement during this step is limited to 0.5 Å from the previous positions, constraining the backbone and preventing the peptide from losing the dominant binding interactions at the P1, P2, and P9 positions. A simple scoring function, reflecting mainly van der Waals interactions is used to evaluate the conformations based on geometric bond matching described by Luo *et al* [52] for protein-ligand docking. Finally, the MM-GBSA procedure is applied (Method S2), including a conjugate gradient minimization procedure and a short molecular dynamics (MD) simulation in which both the peptide and HLA binding groove residues are allowed to be fully flexible. The free binding energy of the final position is then calculated.

### Constructing Three-Dimensional Peptides from Sequence

Choosing the highest resolution nonameric peptides structures bound to HLA-A*02∶01 molecules from the PDB, the peptides were extracted and the side chains atoms at positions 2–9 were stripped off. This resulted in the creation of 13 unique templates, each with a unique residue at P1 (A, F, G, I, K, L, M, N, R, S, T, V, Y). The residues not found in the experimental data (H, D, E, P, W, Q, C) were mapped to the most structurally similar template (based on atomic structure and volumes). *ab initio* peptide models were then constructed using the SCWRL4 side chain modeling software by inputting the template corresponding to the P1 residue [41] and the peptides sequence. In addition, a reference frame consisting of HLA-A*02∶01 backbone atoms was applied to minimize steric clashes. The resulting peptides were then minimized using a short conjugate gradient minimization procedure [53].

### Establishing a Threshold for Binding Peptides

Binding free energy values computed in the MM-GBSA procedure provide a qualitative measure of binding, however they cannot be correlated to quantitative experimental binding constants. Therefore, it is necessary to establish a binding/non-binding threshold for the computed values. To accomplish this, each peptide from our positive and negative peptide pools were docked into the HLA-A*02∶01 molecule. The distribution densities of the calculated ΔG values for the positive (green) and negative (red) peptides are shown in Figure 4c. The significance of the differences between the means of the distributions was calculated using Student’s t-test. The *p* value was estimated at 2.22×10^−16^ for the 95% confidence level, indicating the two distributions are significantly different. A threshold, representing the mean difference between the density peaks for each distribution, is identified as the cutoff. A peptide is considered binding if its ΔG is less than the threshold; otherwise it is considered a non-binder. The accuracy at varying cutoff levels is plotted in Figure 4b.

### Modeling Non-Permissive Residue Substitutions

Structural models of HLA molecules were generated for each observed residue at each non-permissive position, resulting in 35 unique models. The substitutions were manually introduced into a HLA-A*02∶01 starting model [27] using Crystallographic Object-Oriented Toolkit (COOT) [50]. During modeling, high-frequency rotamer orientations were accepted, except where significant steric clashing occurred. A short conjugate gradient minimization was performed on each mutated model [42].

## Supporting Information

Figure S1 **The sequence conservation of peptide datasets.** Sequence logo [54], [55] of the conservation of the 50 peptide data set from the Protein Data Bank (A) and the 5,954 peptide data set from the Immune Epitope Data Bank (B). The sequence logo graphic conveys the amount of sequence conservation at each residue position, with the height of the individual letters representing the information content at each position. The graphic was constructed using the WebLogo webserver (http://weblogo.berkeley.edu/).
(TIF)

Figure S2 **Comparison of crystallographic,** ***ab initio*** **, and docked peptides.** The crystallographic (gray), *ab initio* (blue), and docked (orange) models of the HA-1^Arg^ peptide (*VLRDDLLEA*; PDB id = 3FT4). The peptide is oriented from the side (A), N-termini (B), and top-down view (C). Our methodology utilized an alternate rotamer for the P3 arginine residue that was determined in the crystallographic model, resulting in the poorest performer in our benchmarks. Low occupancy and high B-factors from the experimental data suggest that alternative conformations may be possible for the complex.
(TIF)

Text S1 **Benchmarking Existing p-HLA Complexes.**
(DOC)

Method S1 **IEDB Binding Data Filtering.**
(DOC)

Method S2 **Calculating Binding Free Energies for Peptides Using MM-GBSA.**
(DOC)

Method S3 **Receiver Operator Characteristic Curve.**
(DOC)
