# Supplementary figures and images for: Predicting HLA Class I Non-Permissive Amino Acid Residues Substitutions
Source: PLoS One. 2012 Aug 8;7(8):e41710. doi: 10.1371/journal.pone.0041710 (PMC3414483; doi:10.1371/journal.pone.0041710)

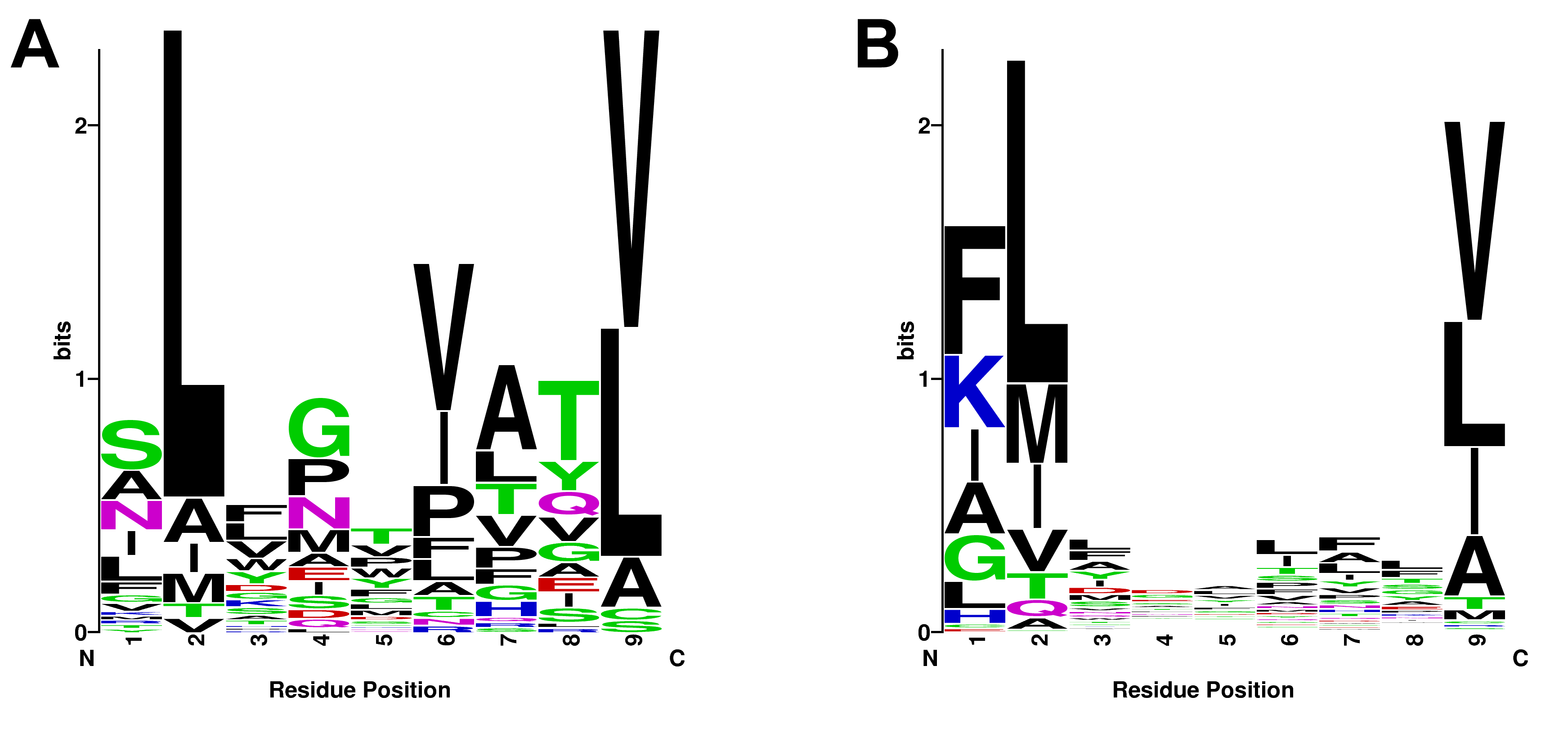

Supplement: Figure S1 — The sequence conservation of peptide datasets. Sequence logo [54], [55] of the conservation of the 50 peptide data set from the Protein Data Bank (A) and the 5,954 peptide data set from the Immune Epitope Data Bank (B). The sequence logo graphic conveys the amount of sequence conservation at each residue position, with the height of the individual letters representing the information content at each position. The graphic was constructed using the WebLogo webserver (http://weblogo.berkeley.edu/). (TIF) [file pone.0041710.s001.tif]

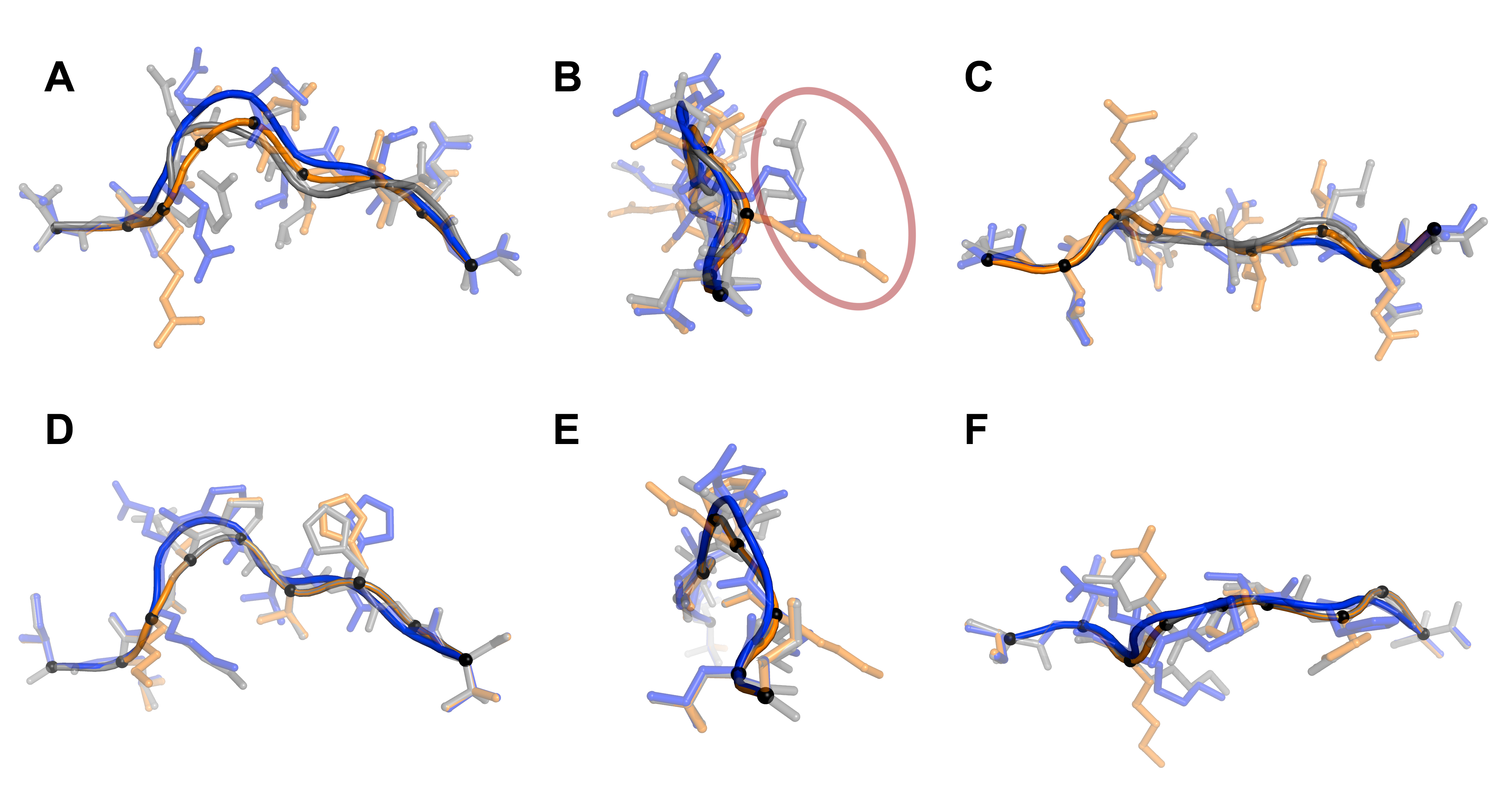

Supplement: Figure S2 — Comparison of crystallographic, ab initio , and docked peptides. The crystallographic (gray), ab initio (blue), and docked (orange) models of the HA-1Arg peptide (VLRDDLLEA; PDB id = 3FT4). The peptide is oriented from the side (A), N-termini (B), and top-down view (C). Our methodology utilized an alternate rotamer for the P3 arginine residue that was determined in the crystallographic model, resulting in the poorest performer in our benchmarks. Low occupancy and high B-factors from the experimental data suggest that alternative conformations may be possible for the complex. (TIF) [file pone.0041710.s002.tif]
