# Supplementary material for: Predicting HLA Class I Non-Permissive Amino Acid Residues Substitutions
Source: PLoS One. 2012 Aug 8;7(8):e41710. doi: 10.1371/journal.pone.0041710 (PMC3414483; doi:10.1371/journal.pone.0041710)
Supplement: Text S1 — Benchmarking Existing p-HLA Complexes. (DOC) [file pone.0041710.s003.doc]

## Text S1: Benchmarking Existing p-HLA Complexes

Our benchmark had one marginal performer, coming in slightly below the 2.5 Å cutoff. HA-1^Arg^ peptide (*VLRDDLLEA*; PDB id=3FT4) is a nonimmunogenic variant of the highly immunogenic HA-1His peptide. HA-1 plays an important role as immunotherapeutic tool in HCT such as in cellular adoptive immunotherapy with HA-1-specific CTLs or in boosting the graft-versus-tumor responses with HA-1 peptide ^1^. Detailed studies showed that the single amino acid difference at position 3 resulting in lack of immunogenicity for HA-1Arg was due to fast dissociation rate due to a combination of structural displacement, steric hindrance and reduced binding network incurred to accommodate the larger and positively charged Arg side chain (P3) in the HLA D pocket ^1^. Upon close inspection, our methodology fails to adopt the strained Arg conformation observed in the crystal structure, opting for a more elongated rotamer (Supplemental Fig. 2). Our rotamer incurs significant steric hindrance, forcing the backbone to be significantly more bowed at the P3 and P4 positions. However, our pose still makes consistent bonds at the anchoring positions and is still within a reasonable overall RMSD. Inspection of the p-HLA complex structure shows low occupancy for the P3 Arg and high B-factors, crystallographic evidence that alternative poses are likely for the bound peptide, some of which may be in closer agreement with our predicted poses. It should also be noted that this peptide has not been benchmarked in any previously reported studies.
