# Supplementary material for: Predicting HLA Class I Non-Permissive Amino Acid Residues Substitutions
Source: PLoS One. 2012 Aug 8;7(8):e41710. doi: 10.1371/journal.pone.0041710 (PMC3414483; doi:10.1371/journal.pone.0041710)
Supplement: Method S1 — IEDB Binding Data Filtering. (DOC) [file pone.0041710.s004.doc]

# Supporting Information

## Methods S1: IEDB Binding Data Filtering

The IEDB provides guidelines to qualitatively interpret the IC50 values as “negative” (IC50>500 nM), “positive-low” (IC50>500 nM), “positive-intermediate” (50nM<IC50<500 nM), and “positive-high” (IC50<50 nM). In some cases a “positive” designation is assigned to peptides, with IC50 values ambiguously ranging from 0.25 to 75000 nM.

Inspection of the data showed some conflicting activity/experimental relationships relating to the “positive” binder nomenclature. For example, the peptide *FKDLFVVYR* had an IC50 of 500 nM, but is classified as a “positive” binder. Another example was *AIAKAAAAV*, which was in the database twice, one instance with an IC50 value of 667 nM and qualitative assessment of “negative” and once with an IC50 value of 79 nM and qualitative assessment of “positive-intermediate”. All peptides whose activity could not be ambiguously reconciled were omitted. In addition, only peptides originating from *H. sapiens* were considered. The resulting data set had 3294 negative and 2660 positive peptides. Of the positive peptides, 371 were “positive-low”, 967 “positive-intermediate”, and 1322 were “positive-high” binders.
