# Supplementary material for: Predicting HLA Class I Non-Permissive Amino Acid Residues Substitutions
Source: PLoS One. 2012 Aug 8;7(8):e41710. doi: 10.1371/journal.pone.0041710 (PMC3414483; doi:10.1371/journal.pone.0041710)
Supplement: Method S2 — Calculating Binding Free Energies for Peptides Using MM-GBSA. (DOC) [file pone.0041710.s005.doc]

## Method S2: Calculating Binding Free Energies for Peptides Using MM-GBSA

The binding free energy was calculated using the MM-GBSA method [1]. This method utilizes molecular mechanical interaction energies with solvation terms based on implicit solvation models. It was shown that explicit waters did not significantly affect the prediction success of modeled p-HLA complexes [2].

The advantages of MM-GBSA are better representation of electrostatic interactions, ligand and protein desolvation energies, and relaxation of the peptide-protein complex. The binding free energy (∆Gbinding) is calculated as:

∆Gbinding = Gp−HLA − GHLA − Gpeptide

∆Gmolecule = ∆EM M + ∆GGB + ∆Gnonpolar − T∆S

where EMM is the molecular mechanical energy, T∆S is the entropy contribution for solvation. The free energy of the p-HLA complex (Gp−HLA), the free HLA-A molecule (GHLA), and the free peptide (Gpeptide) are calculated from snapshots taken from the molecular dynamics (MD) trajectory.

The protein and peptides were prepared using the antechamber suite of programs to create input files that could be read by Leap to generate the parameter and topology files for AMBER. AMBER ff94 parameters were assigned to all protein and peptide atoms. The protonation states of histidine residues were predicted based on their close neighbors. The GB model corresponding to igb=5 in the AMBER program was used. The surface area term was calculated using the LCPO model. A nonbonded cutoff of 18 Å was used for the calculations.

The structures were subjected to 100 steps of conjugate gradient minimization, 3000 steps of MD simulation with a 1-fs time step at a temperature of 300K, followed by 100 steps of minimization. During the minimization and MD, the peptide and all solvent accessible residues in the binding groove were allowed to move. To expedite the scoring process, we calculated the energy of the apo HLA molecule once, and used this energy as a constant term during the subsequent energy evaluations for the rest of the peptides in the data set. This procedure is described for protein-ligand complexes in [3].

##

1. Kollman PA, Massova I, Reyes C, Kuhn B, Huo S, et al. (2000) Calculating structures and free energies of complex molecules: combining molecular mechanics and continuum models. Acc Chem Res 33: 889-897.

2. Fagerberg T, Cerottini J-C, Michielin O (2006) Structural prediction of peptides bound to MHC class I. J Mol Biol 356: 521-546.

3. Graves AP, Shivakumar DM, Boyce SE, Jacobson MP, Case DA, et al. (2008) Rescoring docking hit lists for model cavity sites: predictions and experimental testing. J Mol Biol 377: 914--934.
