# Supplementary material for: Predicting HLA Class I Non-Permissive Amino Acid Residues Substitutions
Source: PLoS One. 2012 Aug 8;7(8):e41710. doi: 10.1371/journal.pone.0041710 (PMC3414483; doi:10.1371/journal.pone.0041710)
Supplement: Method S3 — Receiver Operator Characteristic Curve. (DOC) [file pone.0041710.s006.doc]

## Method S3: Receiver Operator Characteristic Curve

Docking performance experiments are summarized in a Receiver Operator Characteristic (ROC) curve, where the sensitivity is plotted against its specificity at various significance levels of summed probabilities. In the ROC curve, the x-axis represents the false positive rate, or 1-specificity, which is calculated as 1-TN/(TN+FP), where TN is the number of true negatives and FP is the number of false positives. The y-axis represents the true positive rate, or sensitivity, and is calculated as TP/ (TP+FN), where FN is the number of false negatives. An overall performance measure of a classification test can be calculated by the area under the ROC curve (AUC) [1]. Bound between 0 and 1, an AUC of 1 is indicative of a perfectly accurate classification test, in which all true positives are distinguished from false positives. An AUC of 0.5 corresponds to a random classification test (e.g., a coin flip). The AUC is a combined measure of sensitivity and specificity. ROC plots were prepared using the ROCR package for R statistical software [2].

1. Batalia MA, Collins EJ (1997) Peptide binding by class I and class II MHC molecules. Biopolymers 43: 281-302.

2. Sing T, Sander O, Beerenwinkel N, Lengauer T (2005) ROCR: visualizing classifier performance in R. Bioinformatics 21: 3940-3941.
